# The Decision to Fight or Flee – Insights into Underlying Mechanism in Crickets

**DOI:** 10.3389/fnins.2012.00118

**Published:** 2012-08-21

**Authors:** Paul A. Stevenson, Jan Rillich

**Affiliations:** ^1^Institute for Biology, University of LeipzigLeipzig, Germany; ^2^Institute for Neurobiology, Free University of BerlinBerlin, Germany

**Keywords:** aggression, biogenic amines, octopamine insects, assessment, motivation, experience dependent plasticity, decision-making

## Abstract

Ritualized fighting between conspecifics is an inherently dangerous behavioral strategy, optimized to secure limited resources at minimal cost and risk. To be adaptive, potential rewards, and costs of aggression must be assessed to decide when it would be more opportune to fight or flee. We summarize insights into the proximate mechanisms underlying this decision-making process in field crickets. As in other animals, cricket aggression is enhanced dramatically by motor activity, winning, and the possession of resources. Pharmacological manipulations provide evidence that these cases of experience dependent enhancement of aggression are each mediated by octopamine, the invertebrate counterpart to adrenaline/noradrenaline. The data suggest that both physical exertion and rewarding aspects of experiences can activate the octopaminergic system, which increases the propensity to fight. Octopamine thus represents the motivational component of aggression in insects. For the decision to flee, animals are thought to assess information from agonistic signals exchanged during fighting. Cricket fights conform to the cumulative assessment model, in that they persist in fighting until the sum of their opponent’s actions accumulates to some threshold at which they withdraw. We discuss evidence that serotonin, nitric oxide, and some neuropeptides may promote an insect’s tendency to flee. We propose that the decision to fight or flee in crickets is controlled simply by relative behavioral thresholds. Rewarding experiences increase the propensity to fight to a level determined by the modulatory action of octopamine. The animal will then flee only when the accumulated sum of the opponent’s actions surpasses this level; serotonin and nitric oxide may be involved in this process. This concept is in line with the roles proposed for noradrenaline, serotonin, and nitric oxide in mammals and suggests that basic mechanisms of aggressive modulation may be conserved in phylogeny.

## Aggression and the Decision to Fight or Flee

**Figure 1:**
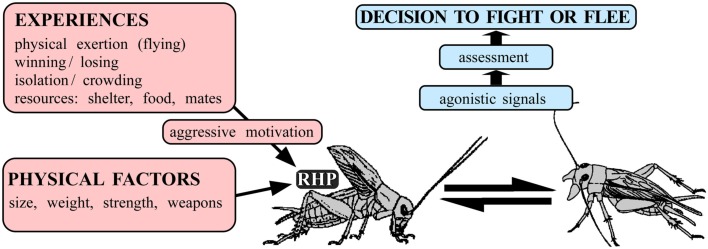
**Factors influencing the decision to fight or flee in intra-specific aggression**. An individual’s prowess at fighting (Resource Holding Potential, RHP) and hence win chances, depends on physical factors (e.g., size) as well as on numerous experiences (e.g., presence and value of resources) that influence aggressive motivation. On confronting a competitor, agonistic signals exchanged during escalating ritualized fighting convey increasingly more accurate information on the individual’s RHP in order to assess whether it would be more opportune to persist in fighting or to flee.

There are many forms of aggression but no uniform definition (see, e.g., Nelson, 2006). In this paper we review insights into how sexually mature insects decide whether to fight or flee when contacting a conspecific of the sex and age under laboratory conditions. Notably, the “struggle for life” is most severe between individuals of the same species, after all, they rival for the same foods, shelter, territory, and sexual partners (Darwin, 1859). This intra-specific aggression is a widespread behavioral strategy in the animal kingdom, which is generally thought of as serving to optimize an animal’s chances of securing limited resources at minimal risk of injury or cost. For aggression to be adaptive, animals must be able to weight up potential benefits and costs in order to “decide” when it would be more opportune to fight or to flee. A variety of hypotheses address how this could be done (c.f. Hurd, 2006). Game theory predicts that aggressive behavior between conspecifics is optimized in “evolutionarily stable strategies” (Maynard Smith and Price, 1973). These are typically stereotyped contests involving the ritualized exchange of agonistic signals, which are thought to convey increasingly more accurate information for assessing the contenders’ “resource holding potential” (RHP), or put simply – win chances (Parker, 1974). The latter will not only depend on physical factors, such as size, strength, and weaponry, but also on metabolic factors (see, e.g., Briffa and Elwood, 2005) and a wide variety of experiences including winning, defeat as well as on the presence, and subjective value of resources at stake such as shelter, territory, food, and mates, that will all determine an animal’s willingness to invest energy in fighting – i.e., its “aggressive motivation” (see Figure 1). These, largely theoretical considerations, provide a neat framework to explain most behavioral observations, such as all else being equal the stronger wins, but that the weaker can prevail when fighting in defense of its offspring. But what are the proximate mechanisms controlling aggression? How do experiences such as resource possession determine “aggressive motivation” and how is this encoded in the nervous system? How do animals “assess” agonistic signals and by which means do they influence the expression of aggressive behavior? Just how exactly do animals make the “decision to fight or flee”?

## Crickets as Model Animals for the Study of Aggression

**Figure 2:**
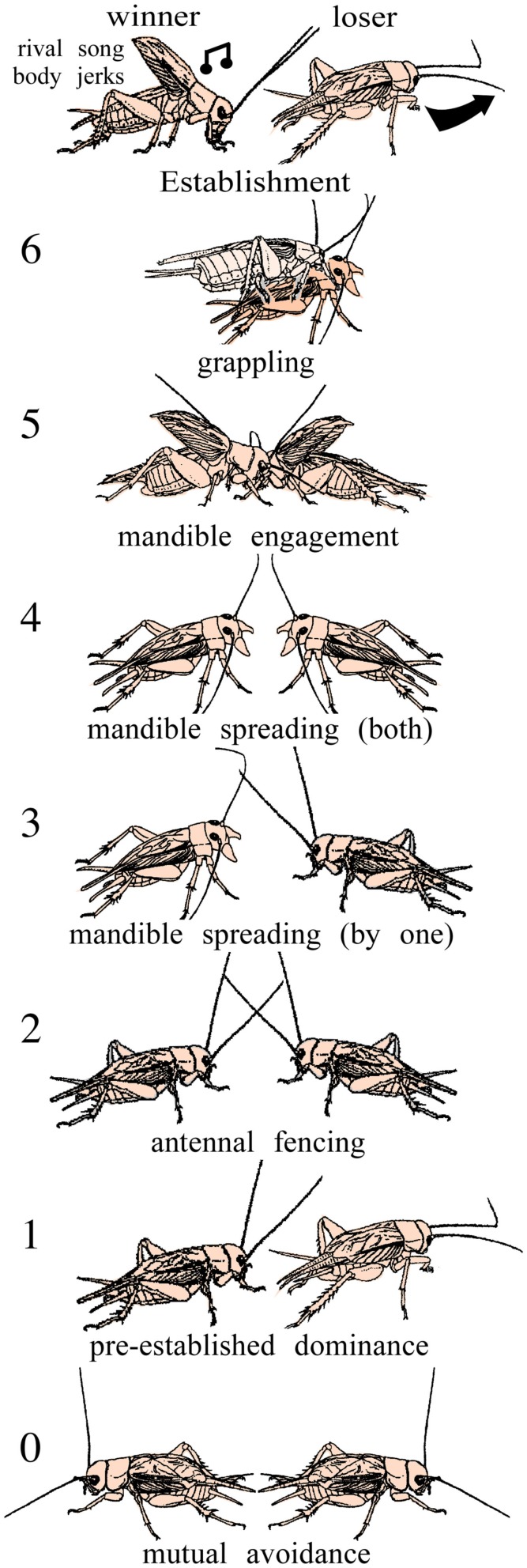
**Stereotyped levels of escalating aggression shown by male adult crickets**. *Level 0* mutual avoidance: no aggressive interaction. *Level 1* pre-established dominance: one cricket attacks, the other retreats. *Level 2* antennal fencing. *Level 3* mandible spreading (by one): one cricket displays spread mandibles. *Level 4* mandible spreading (both): both crickets display spread mandibles. *Level 5* mandible engagement: the mandibles interlock and the animals push against each other. *Level 6* grappling: an all out fight, the animals may disengage, and reengage to bite other body parts. Establishment: the fight can be concluded at any level by one opponent, the loser, retreating, the established winner typically produces the rival song and body jerking movements (modified from Stevenson et al., 2000; Stevenson et al., 2005).

In this review we summarize insights into these questions gained from studies on insects, primarily field crickets (*Gryllus bimaculatus* de Geer). Crickets possess a conveniently sized and comparatively simple, segmentally organized nervous system, and above all have a rich and robust behavioral repertoire (Huber et al., 1989). Their fighting behavior is highly stereotyped and involves a series of easily quantifiable agonistic acts (Figure 2) that can escalate into impressive wrestling contests lasting over a minute and resulting in serious injury (Hofmann and Stevenson, 2000). Fighting establishes clear winners and losers, whereby winners sing a rivalry song, and the losers avoid other males for hours. This is just one example of many illustrating that aggression in crickets is experience dependent. Crickets thus offer the opportunity to investigate nervous mechanisms of context dependent plasticity of social interactions.

## The Decision to Fight – The Role of the Antennae

When two crickets meet they first contact each other with their large moveable antennae and this guides the decision to court, fight, or flee. Species and sex is signaled by the pheromonal signature (Iwasaki and Katagiri, 2008), which induce males to court conspecific females. Females seldom interact, but can fight vigorously in the presence of a male or his courtship song (Rillich et al., 2009). In Drosophila the two sexes adopt different fighting strategies (Nilsen et al., 2004), controlled by the expression of the fruitless gene in specific neurons (Vrontou et al., 2006; Chan and Kravitz, 2007). When male crickets meet they fence vigorously with their antennae and this is both sufficient and necessary to evoke aggressive behavior (Hofmann and Schildberger, 2001). Agonistic responses, such as mandible spreading, can be evoked by simply lashing the antennae with a bristle (Alexander, 1961), or alone by highly volatile male odors (Iwasaki and Katagiri, 2008), which have been identified in fruit flies (Wang and Anderson, 2010). It is thus not surprising that when the antennae are ablated, male crickets frequently court each other but no longer engage each other in fighting (Hofmann and Schildberger, 2001; see also Fernandez et al., 2010 on Drosophila). Striking the antennae directly activates a set of fast conducting descending interneurons (Schöneich et al., 2011), that trigger directed turning responses in some insects (Baba et al., 2010), but their role in cricket aggression remains speculative. Higher brain centers are almost certainly involved in triggering aggression, as indicated by the original finding of Huber (1960) that local electrical stimulation in the vicinity of the mushroom bodies can evoke reproduction of the male rivalry song (Huber, 1960; english summary: Huber et al., 1989).

## The Decision to Fight – The Role of Octopamine

In mammals, the adrenergic/noradrenergic systems are generally accredited with preparing the animal to fight or flee. Insects and other protostomes lack the catecholamine adrenaline and noradrenaline and convert instead the substrate amino acid tyrosine first to tyramine and then to octopamine (c.f. Pflüger and Stevenson, 2005). Recent studies in crickets and fruit flies provide evidence that noradrenaline’s analog octopamine promotes the expression of aggressive behavior in insects.

**Figure 3:**
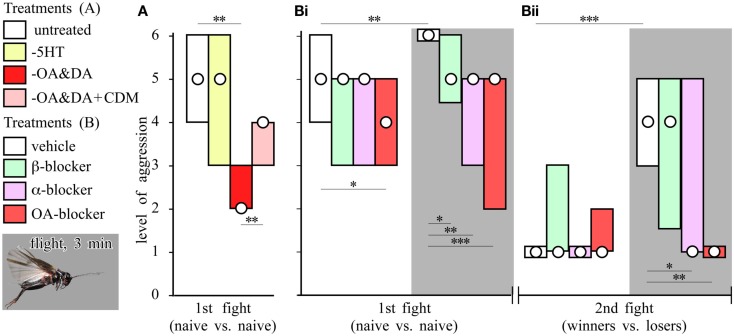
**Aminergic drugs and the effect of flying**. **(A)** Effect of amine depletion on aggression. Bars giving the level of aggression (median, interquartile range) for fights between pairs of socially inexperienced crickets (initial fight) that were either untreated (white bar, n = 24), serotonin depleted (yellow bar, −5HT, n = 27), octopamine/dopamine depleted (red bar, −OA/DA, n = 45) or octopamine/dopamine depleted and treated with the octopamine agonist CDM (pink bar, −OA/DA + CDM, n = 10). **(B)** Effect of aminergic blockers on aggression and the effect of flying (**Bi** initial fight; **Bii** winners vs. losers 15 min later). Before the initial fight the crickets were injected with vehicle (white bars, n = 20), the β-adrenergic blocker propranolol (green bars, n = 19), α-adrenergic blocker phentolamine (violet bars n = 14), or the specific octopamine (OA) blocker epinastine (red bars, n = 20). Seperate groups receiving the same treatments were flown for 3 min just before the initial fight (gray background: vehicle n = 24; propranolol n = 19; phentolamine n = 23; epinastine n = 24). Asterisks denote significant differences between columns indicated (Mann–Whitney *U*-test *p < 0.05, **p < 0.01, ***p < 0.001; simplified from Stevenson et al., 2005).

Fighting behavior in crickets leads to elevated levels of octopamine in the hemolymph (Adamo et al., 1995). Treatment with agents that deplete octopamine and dopamine from the nervous system markedly reduces their aggressiveness and general excitability, which can both be at least partially restored by treatment with the octopamine agonist chlordimeform (CDM), indicating that the defect is most likely due to octopamine depletion (Figure 3A; Stevenson et al., 2000, 2005). Depleting central nervous stores of serotonin, an amine with many functionally antagonistic actions to octopamine (Erber et al., 1993), induces hyperactivity and enhances startle responses, but without affecting aggression. This infers that octopamine’s effect on aggression is selective, and not simply due to increasing general excitability (Stevenson et al., 2005). Similarly in Drosophila, tyramine-β-hydroxylase mutants, which cannot synthesize octopamine and have 10-fold elevated tyramine levels in their brains, have either deficits in aggressive behavior (Baier et al., 2002), or tend to court rather than fight each other (Certel et al., 2007). Hoyer et al. (2008) confirmed that mutant flies lacking octopamine, or octopamine and tyramine, display almost no aggression, and that the defect could be rescued partially by octopamine treatment, or substituting gene function. Furthermore, Zhou et al. (2008) identified a subset of octopaminergic neurons important for aggression in Drosophila and showed that enhancing octopaminergic signaling enhanced aggressiveness.

It is important to stress that octopamine is not essential for actually initiating aggression. For example, crickets lacking octopamine can still exhibit practically all the basic element of aggressive behavior, though usually only when coaxed by repeated antennal stimulation (Stevenson et al., 2000). Taken together, the data suggest that octopamine acts as a modulator that promotes the tendency of insects to fight (Stevenson et al., 2005) and perform agonist acts such as lunging (Hoyer et al., 2008; Zhou et al., 2008) and mandible spreading (Rillich and Stevenson, 2011). This basically corresponds to the modulatory role of octopamine in promoting cholinergic initiating of motor behaviors such as flying (Buhl et al., 2008). As outlined below, experiments on crickets revealed that octopamine mediates the promoting influences of diverse experiences on aggression.

## Experience Dependent Promotion of Aggression

As in mammals and other vertebrates, aggressive behavior in crickets is promoted by a variety of experiences including physical exertion (flying), winning, and the possession of key resources such as food, mates, and shelter (e.g., Alexander, 1961; Dixon and Cade, 1986; Simmons, 1986; Hofmann and Stevenson, 2000; Stevenson et al., 2000, 2005; Nosil, 2002; Killian and Allen, 2008; Rillich and Stevenson, 2011; Rillich et al., 2011). Below we first highlight three illustrative examples, and then summarize evidence showing that octopamine plays a key role in each case (data summarized in Table 1).

**Table 1:** **Effect of various behavioral experiences on the fighting behavior of adult male crickets (control), and how these effects are influenced by selected pharmacological treatments**.

Behavioral experience	Pharmacological treatment and its influence on the effect of behavioral experience					
	Control (vehicle)	OA-agonist (CDM)	OA-blocker (epinastine)	DA or TA blockers	OA-depletion (AMT)	5HT depletion (AMTP)
Control	–	None	Reduced	None	Reduced	None
Losing	Reduced	Restored	None	None	None	None
Flying	Enhanced	–	Blocked	None	Blocked	None
Winning	Enhanced	–	Blocked	None	Blocked	None
Residency	Restored	–	Blocked	None	Blocked	None

**Figure 4:**
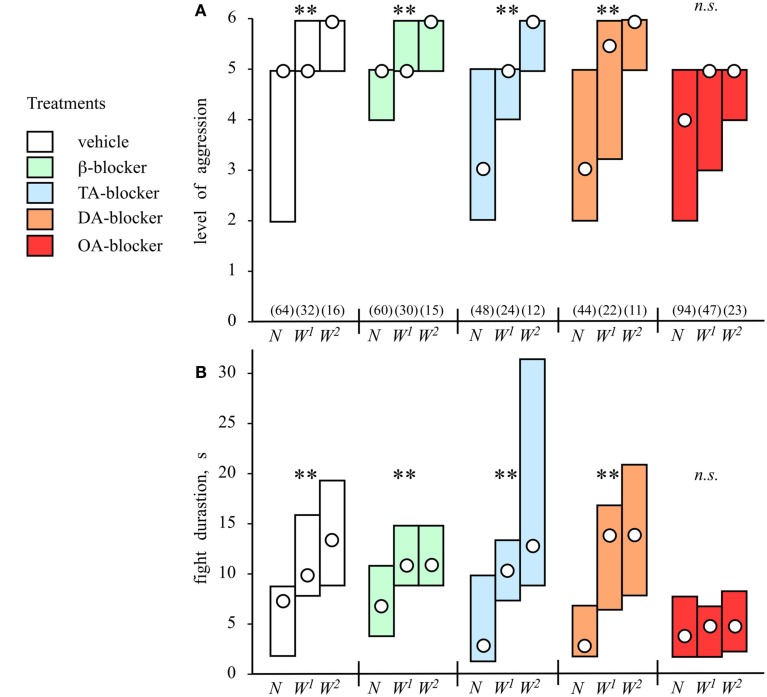
**The winner effect and influences of amine receptor blockers**. Bar graphs giving **(A)** the level and **(B)** duration of aggression (median, interquartile range) for fights between pairs of weight matched male crickets that were socially inexperiences (naive, N), winners of one previous encounter (W^1^), or winners of two encounters (W^2^) for an inter-fight-interval of 5 min. *Pretreatments:* before the initial fight the animals were injected, with: vehicle (white bars), β-adrenergic blocker propranolol (green bars), tyramine (TA) blocker yohimbine (blue bars), dopamine (DA) blocker fluphenazine (brown bars), or octopamine (OA) blocker epinastine (red bars). Numbers in parenthesis in (A) give the pairs for each round, significant differences between tournament rounds are indicated (Kruskal–Wallis one way variance test, **p < 0.01, n.s. not significant; adopted from Rillich and Stevenson, 2011).

**Figure 5:**
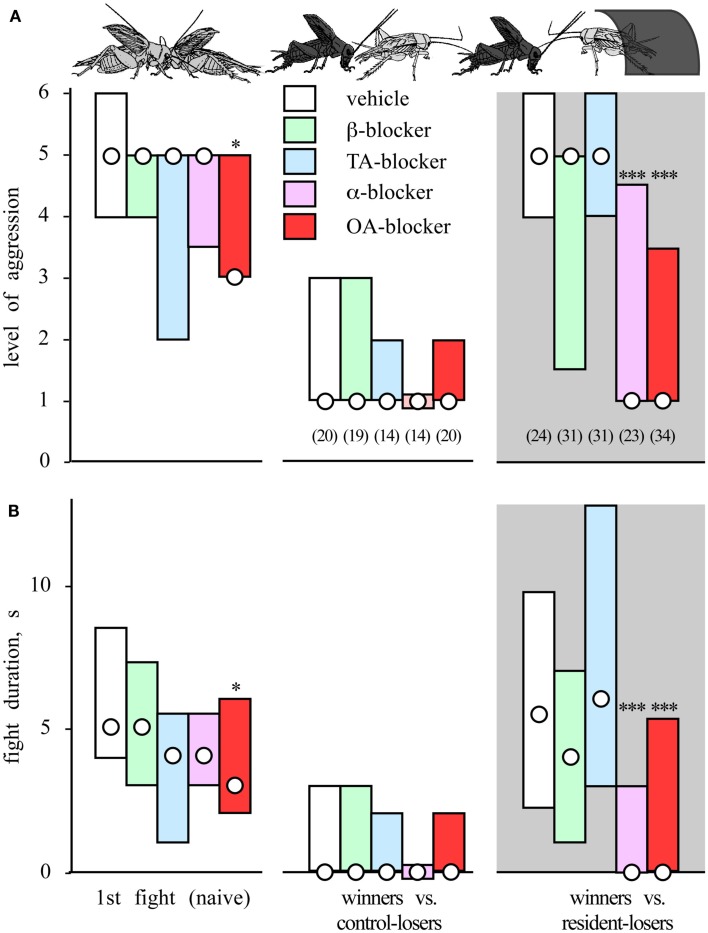
**The residency effect and influences of amine receptor blockers**. Bar charts giving in **(A)** the level of aggression and **(B)** fight duration after residency (median and interquartile range). The crickets were treated prior to the initial fight with either vehicle (white bars), a β-adrenergic blocker (green bars), a tyramine (TA) blocker (blue bars), an α-adrenergic blocker (violet bars), or octopamine (OA) blocker (red bars). The aggressiveness of treated animals was evaluated in an initial fight (naïve) and in a second contact 15 min later before which the losers remained in the arena without a shelter (winners vs. control-losers) or occupied a shelter in the arena (winners vs. resident-losers, gray background). The number of contests evaluated n is given in parenthesis beneath each column, excepting initial fight, which is pooled. Asterisks denote statistically significant differences (Mann–Whitney *U*-test *, **, ***: p < 0.05, 0.01, 0.001 respectively; adopted from Rillich et al., 2011).

*The table summarizes the key results depicted in Figures 3–5 (based on original data from Stevenson et al., 2005; Rillich and Stevenson, 2011; Rillich et al., 2011). OA, octopamine; DA, dopamine; TA, tyramine; 5HT, 5-hydroxytryptamine (serotonin); CDM, chlordimeform; AMT, alpha methyltyrosine; AMTP, alpha methyltryptophan*.

### The effect of flying

Cricket fighting has been a popular pastime in China for centuries (Hofmann, 1996). Surprisingly, “punishing” submissive crickets by shaking and launching them in the air, as recommended by knowledgeable aficionados, significantly increases their aggressiveness, but it is more effective to make the animals fly tethered in a wind stream for a minute or two (Hofmann and Stevenson, 2000). Flown crickets are exceptionally aggressive (Figure 3B), and fight two to three times longer than usual (Stevenson et al., 2005). Moreover, while losers usually avoid other males for hours, flown losers regain their aggressiveness within only 15 min. These effects highlight the impact that motor activity can have on the operation of seemingly unrelated behaviors.

### The winner effect

Winning a conflict makes an individual more aggressive and more likely to win a subsequent encounter in numerous species (reviews: Hsu et al., 2006, 2009; Rutte et al., 2006) including crickets (Khazraie and Campan, 1999; Iwasaki et al., 2006), but little is known of the proximate causes. Recent work implicates androgens as physiological mediators in vertebrates (Oliveira et al., 2009), while in crickets octopamine is involved (Rillich and Stevenson, 2011).

The effect of experiencing successive wins on aggression in crickets has been quantified by staging knockout tournament (Rillich and Stevenson, 2011). With each round, fights between winners of preceding contests become progressively more severe and longer (Figure 4). This winner effect is transient and lasts less than 20 min, which is far shorter than in rodents, and is thus not necessarily associated with learning and memory, as suggested for fruit flies (Yurkovic et al., 2006). But what exactly constitutes a win? When fights between two crickets are interrupted before either experiences an actual win, both contestants become hyper-aggressive in subsequent encounters. However, a winner effect also developed in crickets that experienced an opponent’s retreat prior to any physical interaction. Hence, a winner effect can result both from the physical exertion of fighting, as well as from some non-physical aspect of the actual winning experience.

### Resources and the residency effect

Animals in possession of a key resource, an essentially non-physical experience, are more likely to win disputes against contenders, but it is hotly debated how this is controlled (reviews: Kemp and Wiklund, 2004; Hsu et al., 2006, 2009). For male field crickets, burrows are valuable assets offering shelter from predators and an aid in attracting females, which mate preferentially with burrow owners, and these zealously fight off any intruding male (Alexander, 1961; Simmons, 1986; Rodriguez-Munoz et al., 2011).

Under laboratory conditions, initially submissive crickets become highly aggressive after occupying an artificial shelter and frequently win against an aggressive intruder (Rillich et al., 2011; Figure 5). This residency effect is transient and has a similar time course as the winner effect. It first becomes significant after 2 min of residency, maximal after a 15-min, and declines 15 min after removing the shelter. Hence, the effect does not depend on the initial sensory experience of shelter occupation *per se*. There also seems to be no single feature of the shelter causing the effect. For example, wire shelters, or shelters with a transparent roof are less effective, although darkness alone has no effect. Increased aggressiveness with prolonged residency or territoriality is known in many animal species (Cromarty et al., 1999) and is thought to reflect the increase in value of the resource with time as the animal gathers more information on it or invests increasingly more in it (Bradbury and Vehrencamp, 1998).

### Octopamine dependency

Pharmacological manipulations to evaluate the impact of different biogenic amines on aggression have shown that the effects of flying, winning, and resource possession (residency) are mediated in each case by octopamine. First, the effect of flying can be mimicked by activating the octopaminergic system with CDM, but it is no longer evident in octopamine/dopamine depleted crickets, and it is also selectively blocked by octopamine receptor antagonists (Stevenson et al., 2005; Figure 3B). Similarly, the winner effect is blocked by the selective octopamine receptor antagonist epinastine, but not by β-adrenergic-, tyramine-, or dopamine-receptor antagonists (Rillich and Stevenson, 2011; Figure 4). Finally, the residency effect is prohibited in octopamine/dopamine depleted crickets, while being unaffected by serotonin depletion, and it is selectively blocked by treatment with octopamine antagonists (Rillich et al., 2011; Figure 5).

## Octopamine, Reward, and Aggressive Motivation

The paradoxical question now posed is how experiences as diverse as flying, winning, and resource possession, which encompass extremes of the locomotory and energy expenditure spectrum, can all lead to activation of the octopaminergic system promoting aggression? Activation of the insect octopaminergic system is generally thought to occur under stressful conditions and prepare the animal for a period of prolonged activity, or assist in recovering from increased energy demand (Verlinden et al., 2010). Flying and fighting both lead to a considerable increase in the hemolymph titer of octopamine (Adamo et al., 1995), although the concentration is too low to pass the “blood-brain” barrier and influence aggression (c.f. Stevenson et al., 2005). The increase must result partly from “spill over” from efferent octopaminergic neurons, such as the dorsal and ventral unpaired median neurons (DUM/VUM cells, reviews: Stevenson and Sporhase-Eichmann, 1995; Bräunig and Pflüger, 2001), which innervate skeletal muscles and are excited during flying (Duch et al., 1999), walking (Baudoux et al., 1998), and by a variety of mechanosensory signals (Morris et al., 1999). Although the activation of octopaminergic neurons due to the physical exertion of flying and fighting could explain the effects of these activities on aggression, the argument is less compelling for the influences of winning without fighting and residency. The latter, essentially non-physical experiences, are also unlikely to represent stressful conditions.

As an alternative hypothesis, we propose that all experiences that enhance aggressiveness in crickets do so because they in someway represent a positive, reinforcing, or rewarding experience (Rillich and Stevenson, 2011; Rillich et al., 2011). Physical exercise in mammals, including humans, seems to be equated with reward (Raichlen et al., 2011), and can act as a mood elevator that alleviates symptoms of depression by invoking changes in a variety of neurotransmitter systems including dopamine (Craft and Perna, 2004). Aggression itself also leads to increased activity in dopaminergic pathways and androgen receptor expression in regions of the mammalian brain that mediate motivation and reward (Barron et al., 2010; O’Connell and Hofmann, 2011). Even watching a previous victory can evoke similar effects in humans (Carre and Putnam, 2010). In insects, evidence suggests that reward is signaled by octopamine, rather than dopamine (review: Barron et al., 2010). In honeybees, the nutritional value of food sources may be encoded by octopamine modulating associative reward pathways (Barron et al., 2010). Octopamine conveys reward signals in appetitive learning in honeybees (Hammer and Menzel, 1995), fruit flies (Schwärzel et al., 2003), and crickets (Mizunami et al., 2009). In the honeybee, the activity of even a single identified octopaminergic DUM/VUM-type neuron can substitute for the sucrose reward in an associative learning paradigm (Hammer, 1993). This neuron is one of a group of less than 20 octopaminergic DUM/VUM-neurons occurring in the subesophageal ganglion of honeybees (Schröter et al., 2007) and other insects including crickets (Stevenson and Sporhase-Eichmann, 1995). In Drosophila, a distinct subset of these octopaminergic neurons was shown to be functionally important for expressing aggression (Zhou et al., 2008). Another subset expresses the sex determining factor fruitless, and is involved in mediating the choice between courtship and aggression (Certel et al., 2007, 2010). The function of these neuron types in crickets is unknown.

## The Decision to Flee – The Cumulative Assessment Model

**Figure 6:**
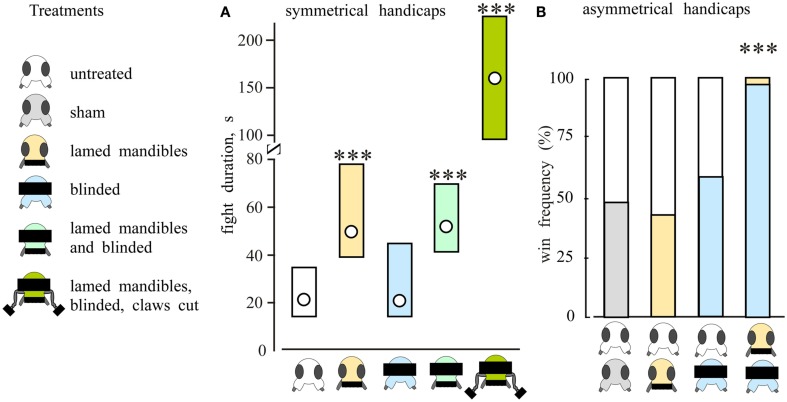
**Handicaps reveals assessment strategy in crickets**. **(A)** Symmetrical handicaps. Bars giving the level of aggression (median, interquartile range) for fights between pairs of crickets that were both (from left to right) untreated (n = 38), had lamed mandibles (n = 26), blinded (n = 19), had lamed mandibles and blinded (n = 26) or lamed mandibles, blinded, and clipped foreleg claws (n = 23). **(B)** Asymmetrical handicaps. Bars giving the win frequencies (%) for handicapped crickets, from left to right: sham vs. untreated (n = 33); lamed mandibles vs. untreated, (n = 45) blinded vs. untreated (*n* = 50), blinded vs. lamed mandibles (n = 35). Statistically significant differences between data sets are indicated [**(A)**, *U*-test; **(B)**, Chi-square; *, **, ***: p < 0.05, 0.01, 0.001 respectively; adopted from Rillich et al., 2007].

For the decision to flee, animals are thought to assess information from ritualized agonistic signals exchanged during fighting. This decision could be based on average differences in signals (Sequential Assessment Model), the total sum of own actions (Energetic War of Attrition Model), or the total sum of opponent actions (Cumulative Assessment Model; c.f. Payne, 1998). Work on crickets revealed that agonistic signals act to reduce the aggressiveness of the receiver, but not the sender (Rillich et al., 2007). For example, pairs of crickets with lamed mouthparts not only fight, they escalate higher and fight longer than sham treated crickets (Figure 6A). Fights became progressively longer the more the animals were handicapped, lasting minutes rather than seconds for example between opponents with lamed mandibles, blackened eyes, and clipped foreleg claws to limit body flipping. Furthermore, whereas “blinded” crickets, or crickets with lamed mouthparts fought non-handicapped crickets with almost unaltered win chances, the blinded crickets practically always (98%) defeated crickets with lamed mouthparts (Rillich et al., 2007; Figure 6B). These findings are fully conform with predictions of the cumulative assessment model postulated by Payne (1998). We suggest, in accord with this model, that Mediterranean field crickets persist in fighting until the sum of the perceived adversary’s actions accumulated during fighting surpasses some threshold to flee. Hence, the blinded cricket persists because it receives no visual and limited physical input from an opponent with lamed mandibles, whereas the latter accumulates the full brunt of his adversaries actions and becomes the first to flee. This model also accommodates the effects of physical disparities such as size strength and weaponry on fight outcome (see, e.g., Judge and Bonanno, 2008; Hall et al., 2010), since an animal with any physical advantage will have a greater sensory impact on its opponent, which in turn increments opponent agonist signals more rapidly, and is thus more likely to flee first. It also fits our personal observation that crickets often fight on even after serious injury, such as losing part of a leg or antenna, only to retreat seconds later for no obvious reason.

## The Decision to Flee – Candidates for Its Control

It is not known how the sensory information of various modalities conveyed by an opponent’s agonistic signals is summated in the nervous system and how this triggers a cricket to retreat. Whatever its cause, defeat results in longer-term submissiveness in many animal species (reviews: Rutte et al., 2006; Hsu et al., 2009). This loser effect lasts over 3 h in crickets, during which time they avoid contact to other males, even if unfamiliar (Hofmann and Stevenson, 2000). Nonetheless, losers are still potentially aggressive, since they will fight for example when their eyes are blackened, a manipulation that effectively eliminates the visual sensory impact of the approaching opponent (Rillich et al., 2007). Accordingly, the reduced tendency of losers to fight seems to be due to an increased tendency to flee, rather than reduced aggressiveness *per se*. Supporting this idea, retreat, and the loser effect appears not to result from depressed octopaminergic signaling. For one, octopamine levels are similar in winners and losers (Adamo et al., 1995). Furthermore, the octopamine agonist chlordimeform (CDM), which binds almost irreversibly to octopamine receptors (c.f. Evans, 1985), can restore aggression in losers, but cannot protect them from actually losing, and subsequently behaving submissively for a short time, after which their aggression is once again restored under the continued influence of CDM (Stevenson et al., 2005). There must, therefore, be some opposing control mechanism, which could involve the following neuromodulators.

### Serotonin

The actions of octopamine in arthropods are often functionally antagonized by serotonin (Erber et al., 1993). Serotonin’s role in insect aggression is, however, unclear. In crickets, serotonin depletion induces hyperactivity and enhances startle responses, but without affecting aggression (Stevenson et al., 2000, 2005). Supporting this, Baier et al. (2002) found that aggression in Drosophila is unaffected when serotonin synthesis is either disrupted, or its level elevated by treatment with serotonin’s precursor (5HTP). In contrast, Dierick and Greenspan (2007) observed that 5HTP promotes aggression in fruit flies, and Alekseyenko et al. (2010) using genetic manipulation report that activating serotonergic neurons resulted in flies that escalated faster and fought fiercer, while disrupting serotonergic transmission yielded flies with reduced fighting ability (see also Dyakonova et al., 1999 on crickets). These conflicting findings may be due to differences in behavioral protocols together with difficulties in dissecting out differential effects of serotonin operating via different receptor subtypes. Johnson et al. (2009) for example, found that pharmacological activation of 5HT2-type receptors reduced total aggression in Drosophila, and conversely that activating 5HT1A-type receptors increased it.

In mammals, different serotonin receptor subtypes also seem to influence different aspects of the total aggressive behavioral repertoire (de Boer and Koolhaas, 2005). This and other findings now challenge the dogmatic view of serotonin acting simply to suppress aggression. Currently, serotonin in mammals is thought to limit impulsivity (review: Nelson and Trainor, 2007) or promote the drive to withdraw (Tops et al., 2009), rather than suppress aggression *per se*. We envisage an analogous scenario in insects, since it fits our observations in crickets that losers have an increased tendency to flee, rather than suppressed tendency to fight. The current evidence is however limited. While serotonin seems to depress escape responses in aggressive crickets (Dyakonova et al., 1999), losers are claimed to exhibit enhanced escape behavior due to lower serotonin levels (Murakami and Itoh, 2001). Similarly in crayfish, the effects of serotonin on escape and body posture change with social status due to a shift in the relative expression of different serotonin receptor subtypes to a pattern more appropriate for the new status (Edwards and Spitzer, 2006; Cattaert et al., 2010). Finally in locusts, visual, and tactile inputs from conspecifics induce the release of serotonin, which promotes social tolerance (Anstey et al., 2009). It is thus conceivable, that serotonergic pathways are activated in crickets by the perceived agonistic signals of an opponent during fighting.

### Peptides

The expression of aggression in insects is also influenced by the action of neuropeptides. In crickets, treatment with the opiate antagonist naloxone elevates aggressiveness in losers, without affecting winners, or socially naive animals (Dyakonova et al., 2002) and in Drosophila aggression is increased following genetic silencing of circuitry employing neuropeptide-F, the invertebrate homolog of neuropeptide-Y (Dierick and Greenspan, 2007).

### Nitric oxide

Aggression in mammals is suppressed by the action of the gaseous modulator nitric oxide (NO), at least partly by influencing serotonergic signaling (Nelson and Trainor, 2007), but its role in insect aggression needs clarification. Dyakonova and Krushinskii (2006) report that treatment with an NO-synthesis inhibitor prohibits the aggression promoting effects of flying in crickets, indicating that NO enhances aggressiveness. Iwasaki et al. (2007) in contrast, report that inhibiting NO-synthesis relieves the loser effect, but has no effect on socially naive crickets. On going work indicates that disrupting the NO/cGMP pathway causes socially naive crickets to persist longer at fighting (Stevenson, 2011; Stevenson, in preparation), suggesting that accumulating NO may be involved in triggering the decision to flee.

## A Relative Threshold Model for the Fight or Flee Response

**Figure 7:**
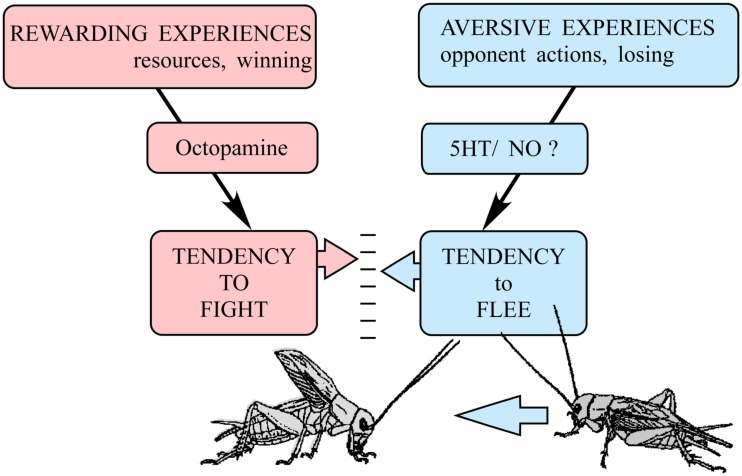
**A relative threshold model for the decision to fight or flee in crickets**. Rewarding experiences (winning, resource possession) promote the tendency to fight via the action of octopamine. In this respect, octopamine can be regarded as representing the motivational component of aggression. Aversive experiences accumulated during fighting (opponent’s agonistic signals, losing) promote the tendency to flee via actions of other neuromodulators, which probably include serotonin (5HT), nitric oxide (NO), and selected peptides. In accord with the cumulative assessment hypothesis (Payne, 1998), a cricket will fight and persist as long as the perceived sum of rewarding experiences (motivation) exceeds the sum of accumulated aversive fighting experiences, but flee the moment the latter is greater. (adopted from Simpson and Stevenson, 2012).

We propose that the decision to fight or flee could be accounted for in crickets by simply modulating the initiation thresholds for these two opposing behaviors relative to each other (Figure 7). As argued above, experiences evaluated as being in someway rewarding (winning, resource possession) promote the tendency to fight to a level determined by the modulatory action of octopamine. Accordingly, octopamine can be considered as representing the motivational component of aggression. Opposing this, and in accordance with the cumulative assessment hypothesis (Payne, 1998), aversive experiences, i.e., the opponent’s agonistic signals, trigger the tendency to flee when the accumulated sum surpasses a set level. It appears likely that serotonin, nitric oxide, and selected peptides are involved in integrating agonistic signals for the decision to flee. This model is in line with the roles proposed for noradrenaline, serotonin, and nitric oxide in mammals (Tops et al., 2009), suggesting that basic mechanisms of aggressive modulation may be conserved in phylogeny. However the principle actions of serotonin and octopamine on aggression are apparently reversed in crustaceans (Kravitz and Huber, 2003), so they do not fit into this schema. Regardless of the actual modulators involved, the relative threshold model would allow the animal to optimally adapt its aggressive behavior toward an opponent by taking into account both physical disparities as well as experience dependent disparities in aggressive motivation.

## Conclusion and Future Directions

The brains of insects may be comparatively simple in terms of neuron number, they nonetheless have the integrative power to sculpture social interactions of a complexity approaching that of our own (Simpson and Stevenson, 2012). Insects are thus ideal models for investigating how animals make appropriate adaptive behavioral choices. As such, they should provide insights for the currently en-vogue discipline of neuroeconomics (Glimcher et al., 2009), which seeks to determine how costs, and benefits are represented in the brain for optimizing decision-making. The studies on crickets outlined here illustrate that insects have the capacity to compute potential rewards and costs of aggression for making the adaptive behavioral decision to fight or flee on confronting a competitor. They achieve this it seems quite simply, by exploiting the powers of neuromodulation, primary using biogenic amines, which act at the interface between the animal’s social environment and central brain circuits (Simpson and Stevenson, 2012).

It has been shown that the tendency to fight in insects is promoted by the amine octopamine, the analog of noradrenaline. Rather than acting as a releaser of aggression, or simple “arousal” agent, octopamine appears rather to function as a selective neuromodulator that mediates the aggression promoting effect of experiences, including physical exertion, winning, and the possession of resources. In this respect octopamine represents the motivational component of aggression that drives the tendency of a cricket to fight. In correspondence with the envisaged role of dopamine on aggression in mammals (reviews: Barron et al., 2010; O’Connell and Hofmann, 2011), we propose that experiences that promote aggression in crickets are evaluated as being in someway rewarding. What we now need to know is whether octopamine encodes the actual value of a resource for the decision to fight. This seems feasible considering that foraging honey bees appear to exploit the octopaminergic system to report the quality of discovered food sources (Barron et al., 2009).

The decision to flee, on the other hand, appears to be controlled in line with predictions of the cumulative assessment hypothesis (Payne, 1998) in that crickets persist in fighting until the sum of the perceived adversary’s actions surpasses some threshold to flee. Defeated crickets have a reduced tendency to fight, but are still potentially aggressive, indicating that losing increases the tendency to flee, rather than reduce aggressiveness *per se*. Future studies must now be directed toward discovering how information from an opponent’s agonistic signals are summated in the nervous system and how this could promote the drive to withdraw. The first step must be to evaluate the extent to which serotonin, nitric oxide, and possibly peptides are involved in this process. At present we also do not know how the decision to flee is influenced by the energetic costs of physical fighting, which is high in crickets (Hack, 1997). In hermit crabs it appears that the depletion of energy reserves and accumulation of harmful by-products are cues for the decision to give up (Briffa and Elwood, 2005). In crickets we predict (in accord with the cumulative assessment hypothesis), that accruing metabolic costs may correlate with abating physical fitness, and hence lowered sensory impact on the opponent, which will hence tend to persist longer and be less likely to flee first.

In conclusion, we propose that the decision to fight or flee in crickets is controlled by the action of separate neuromodulator system that set the relative behavioral thresholds for these opposing behaviors. A simple threshold mechanism also has the power to control sophisticated collective decision-making in eusocial insects (Robinson et al., 2011). The model we propose is in line with the roles envisaged for noradrenaline, serotonin, and nitric oxide in mammals (Tops et al., 2009), but differs to that in crustaceans in which the principle actions of serotonin and octopamine are apparently reversed (Kravitz and Huber, 2003).

While some progress has been made in elucidating the nervous centers and neuroanatomical pathways underlying aggression in rodents and non-human primates (review: Nelson and Trainor, 2007), we are far from knowing this in crickets, despite their reputedly more accessible and comparatively simpler nervous system. Individual octopaminergic cells involved in the neuronal representation of rewarding qualities have been identified in the honeybee as individuals of the group of ascending DUM/VUM cells in the subesophageal ganglion (Hammer, 1993). Neurons of this class may also be involved in the expression of aggression in fruit flies (Zhou et al., 2008). They also occur in crickets and other orthopterans (Stevenson and Sporhase-Eichmann, 1995), but their functions are largely unknown. In the insects investigated, individual DUM/VUM neurons were found to invade all major brain neuropils, including the mushroom bodies, a region where focal electrical stimulation was shown to elicit discrete elements of aggressive behavior in crickets more than 50 years ago (Huber, 1960). We now need to discover the synaptic connectivity of the ascending octopaminergic DUM/VUM cells in crickets, and in particularly the locality and types of receptors they activate. These and other aminergic neurons are often equipped with a host of co-transmitters, including nitric oxide, amino acids, and peptides (e.g., Bullerjahn et al., 2006), but it is not known under which behavioral circumstances co-transmitters are released, nor how they affect modulation at their targets. Finally, on a topic we have brushed past, aggression can have longer-term changes on the operation of the nervous system than those discussed here. Agonistic behavior can trigger neurogenesis (Ghosal et al., 2009) and FOS-like protein expression in the male cricket brain (Ghosal et al., 2010), but it is not know whether this leads to changes in behavior. A hint of the complexities involved is given by the finding that aggressive behavior in Drosophila is affected by over 50 novel genes with widespread pleiotropic effects (Edwards et al., 2009).

## Conflict of Interest Statement

The authors declare that the research was conducted in the absence of any commercial or financial relationships that could be construed as a potential conflict of interest.
